# Anti-tumour necrosis factor-alpha agent therapy, compared with conventional therapy, reduces the relapse of uveitis in patients with behçet’s disease: A systematic review of controlled trials

**DOI:** 10.3389/fphar.2022.912906

**Published:** 2022-08-19

**Authors:** Xinwei Zhou, Xianghui Shi, Yanxia Ren, Tingting Yan, Qiao Ye

**Affiliations:** ^1^ Department of Vascular Surgery, The Second Affiliated Hospital of Jiaxing University, Jiaxing, China; ^2^ Department of Rheumatology, The Second Affiliated Hospital of Jiaxing University, Jiaxing, China

**Keywords:** anti-tumour necrosis factor-alpha, uveitis, behçet disease, systematic review, control trials

## Abstract

**Purpose:** Anti-tumour necrosis factor-alpha (TNF-α) agents are often used for Behçet’s disease (BD) in clinical practice, but they have not been validated by a high level of evidence. We systematically reviewed published controlled trials to investigate the efficacy and safety of anti-TNF-α therapy and summarize the efficacy of anti-TNF-α therapy relative to the available therapeutic options.

**Methods:** A systematic database search was conducted (PubMed, Embase and Cochrane) using specific search terms. All controlled studies of anti-TNF-α treatment of BD patients prior to December 2021 were included. Single-arm studies were excluded. The decision of whether to incorporate data into the meta-analysis or summarize the data by qualitative synthesis was based on the results of the literature screening.

**Results:** Of 4389 screened studies, 13 (total 778 patients) were included in accordance with our retrieval strategy, comprising 1 randomized controlled trial, 1 prospective study, 10 retrospective studies, and 1 multicentre open-label study. Ten studies (76.9%) involved Behçet’s uveitis (BU), 1 involved intestinal BD, and the other studies had undefined subtypes. Subgroup reviews were conducted according to the control drug. Four studies involving 167 participants reported relapse rates. Meta-analysis of three of these studies demonstrated that, compared with traditional immunosuppressant (TIS) therapy, anti-TNF-α therapy reduced the relapse rates in patients with BU. In targeted drug comparison studies, the efficacy appeared to be similar between the anti-TNF-α agent and interferon in BU patients. The rates of adverse events were comparable between a variety of different therapeutic controls. Serious adverse events were not observed in 53.8% (7/13) of the studies.

**Conclusions:** Compared with TIS therapy, anti-TNF-a therapy reduces the relapse of uveitis in patients with BD. However, the evidence regarding anti-TNF-α therapy is very limited for the full spectrum of BD subtypes, which calls for caution.

## 1 Introduction

Behçet’s disease (BD) is a multisystem inflammatory disease of unknown aetiology characterized by recurrent oral and/or genital aphthous ulcers and skin lesions, accompanied by multiple organ involvement. The pathogenesis of BD is unknown; however, there is evidence pointing to numerous dysregulated cytokines in patients with BD that can be clinically correlated with the clinical phenomenon (Akdis et al., 2016; Leccese and Alpsoy, 2019; Tong et al., 2019; Perazzio et al., 2020). Tumour necrosis factor (TNF)-α, a well-known proinflammatory cytokine with a wide range of biological functions, is secreted primarily by monocytes and macrophages (Salomon et al., 2018). Studies have shown that serum TNF-α expression is abnormally elevated in patients with BD (Neves and Spiller, 2013; Abdolmohammadi and Bonyadi, 2017) and is considered a key element in the inflammatory pathway of BD (Aderem and Ulevitch, 2000; Gül, 2015; Van der Houwen et al., 2020). In recent years, research on the immunological mechanism of BD has laid a foundation for the development of targeted therapy.

Data from a systematic review showed that the use of anti-TNF-α therapy to achieve remission in BD is a promising strategy (Arida et al., 2011). In addition, a few previous meta-analyses have reported the therapeutic value of anti-TNF-α in various complications of BD (Hu et al., 2020; Zhang et al., 2022), such as Behçet’s uveitis (BU) or intestinal BD; however, these papers all share a common defect. That is, these included studies overwhelmingly were clinical single-arm studies with no comparison arms and no reliable quality assessment. When single-arm, noncomparator studies make up most of the available evidence, systematic reviews and meta-analyses are at significant risk of bias. At the same time, the conventional drugs may be able to achieve a level of efficacy. The choice between cost-effective conventional drugs or targeted therapy needs to be weighed more carefully. Therefore, it is important to clarify the status of anti-TNF-α therapy among the multiple available therapeutic options. Based on the above considerations, we conducted a systematic review based on controlled trials to summarize the evidence of the efficacy and safety of anti-TNF-α therapy for all BD subtypes.

## 2 Materials and methods

### 2.1 Selection criteria

Studies were eligible for inclusion if they met all of the following criteria: 1) full-text publications; 2) controlled study with interventional (randomized or nonrandomized) or observational (prospective, retrospective or case–control) design; and 3) participants who met the diagnostic criteria of BD, which could include any of the major subtypes, such as BU, intestinal BD, neuro-BD, and vasculo-BD. All patients received anti-TNF-α therapy at least once. The anti-TNF-α agents included adalimumab, infliximab, golimumab, etanercept and certolizumab, The drug-related attributes are shown in Table 2.

Exclusion criteria were as follows: 1) studies only available in abstract form; 2) single-arm studies, case reports, case series, conference literature, letters, reviews and meta-analyses; 3) repeat publications (the latest publication was chosen in sush cases); and 4) inability to extract the data from the paper.

### 2.2 Data sources and search strategy

We conducted a systematic search in MEDLINE (from January 1950 to 17 December 2021) accessed via PubMed, Embase (from January 1980 to 17 December 2021) and the Cochrane Library (to 17 December 2021). The search strategy was constructed by using both a controlled vocabulary (namely, MeSH in MEDLINE and EMTREE in Embase) and a wide range of free-text terms, and all the retrieval strategies were determined after multiple preretrievals. The terms “Behçet’s Disease”, “Tumour Necrosis Factor-alpha”, “Adalimumab”, “Infliximab”, “Golimumab”, “Etanercept” and “Certolizumab” were employed. Publications were limited to those written in English.

### 2.3 Literature screening and data extraction

Two review authors (X.Z. and T.Y.) independently screened the literature according to the predetermined inclusion and exclusion criteria. The review authors excluded literature that clearly did not meet the inclusion criteria after reading the titles and abstracts. They then read the full text of all potentially relevant studies. They designed a data extraction table into which they independently extracted the study data. They resolved disagreements about the extracted data by consensus or with a third review author (Q.Y.). We sought to obtain supplementary files if the data were incomplete.

The extracted data included the following: 1) test method and basic information on the two groups of subjects; and 2) Study design, nation, study time, sample size, follow-up time, intervention measures, mean age, sex, treatment, and indicators of response, study quality, etc. Outcomes included 1) visual acuity (VA): best-corrected visual acuity (BCVA) assessed using the Snellen test or VA logarithm of the minimum angle of resolution (logMAR); 2) macular thickness (MT): MT was defined as the vertical length between the macular fovea and its corresponding point in the retinal pigment epithelium; 3) number of relapses: relapse was defined as a new flare-up or aggravation of uveitis; 4) response rates: complete response, partial response, remission rates and visual improvement; 5) adverse events (AEs): pay attention to serious adverse events (SAEs), and infectious AEs. Those not specified in the original paper were determined by the review authors based on the description in the paper.

### 2.4 Risk of bias assessment and data synthesis

The methodological quality of the included studies was evaluated according to the quality evaluation tools recommended by the Cochrane Handbook 5.1.0 (Higgins and Green, 2011). Disagreements were resolved by discussion with a third review author (Q.Y.). The decision of whether to incorporate the data into the meta-analysis or to summarize the data in a qualitative synthesis was based on the results of the literature screening. Dichotomous variables are expressed as absolute frequency (percentage), and continuous variables are described as median [range or interquartile range (IQR)]. Meta-analysis was performed using RevMan 5.3 software provided by the Cochrane Collaboration. The odds ratio (OR) and 95% confidence interval (CI) were used for dichotomous variables. The mean difference (MD) and 95% CI were used for continuous variables. It is permissible to use a formula to convert the median (quartile range (IQR) or range) to the mean standard deviation (SD) (Wan et al., 2014; Luo et al., 2018). The χ2 test was used for heterogeneity analysis. Fixed-effects or random-effects models were used according to the heterogeneities (I^2^ < 50%, fixed-effects models; I^2^ > 50%, random-effects models). In contrast, if the data could not be combined, qualitative conclusions were made, and meta-analysis was only used as supplementary evidence.

## 3 Results

### 3.1 Literature search

**FIGURE 1:**
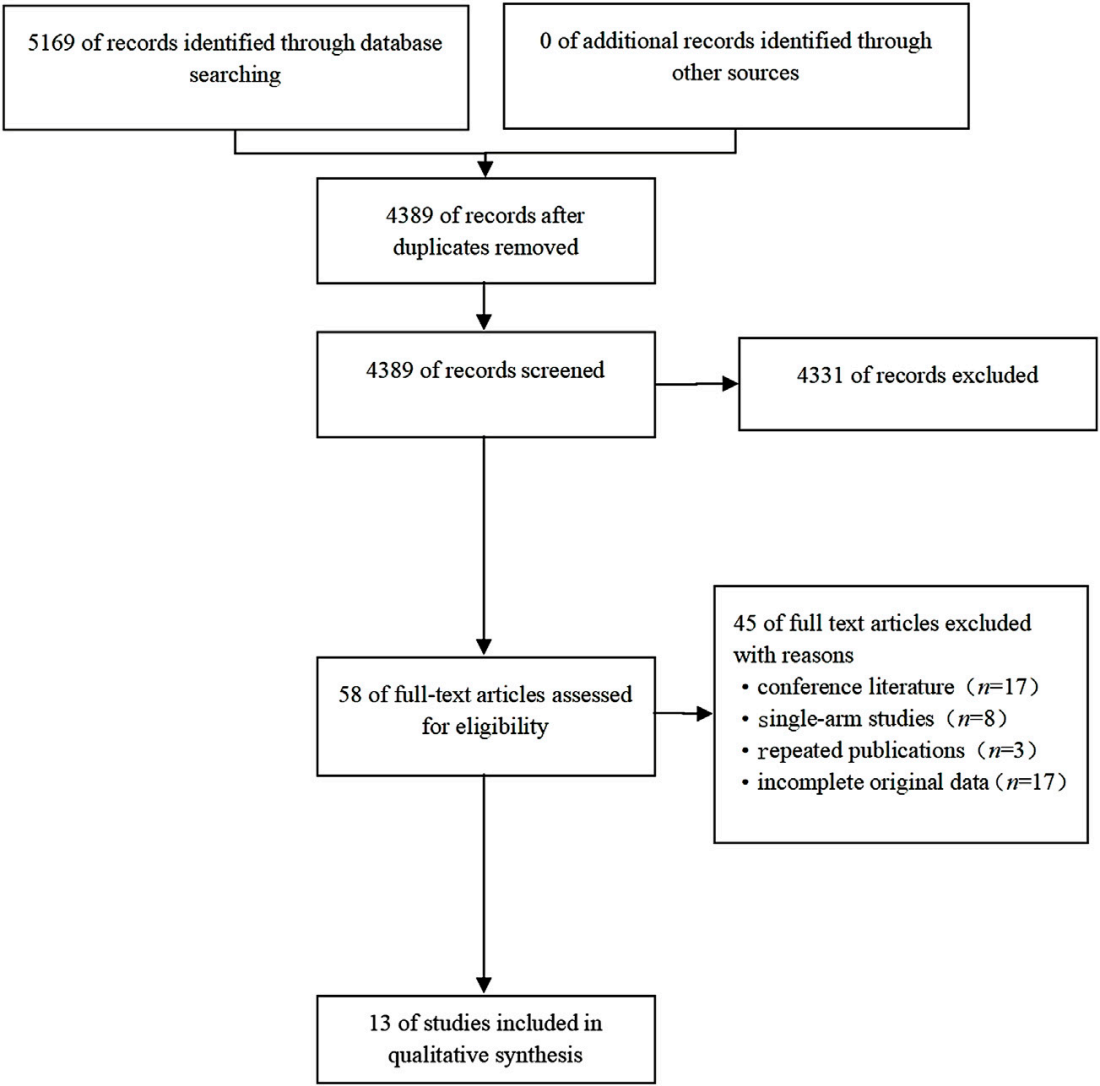
Flow chart of the search strategy.

We initially identified 5169 articles from the following databases: PubMed (*n* = 1045), Embase (*n* = 3246) and the Cochrane Library (*n* = 878). We identified 0 records from other sources. We retrieved 4389 references after duplicates were removed. Ultimately, we were left with 58 articles for further assessment by reviewing the titles and abstracts. After a careful assessment of the full text in the included studies, 45 articles were removed due to nonextractable results and the outcome of interest. Finally, 13 eligible studies were included in accordance with our retrieval strategy (Melikoglu et al., 2005; Tabbara and Al-Hemidan, 2008; Yamada et al., 2010; Markomichelakis et al., 2011; Ma et al., 2014; Vallet et al., 2015; Fabiani et al., 2017; Atienza-Mateo et al., 2019; De Simone et al., 2020; Kunimi et al., 2020; Yalçindag and Köse, 2020; Yang et al., 2021a; Yang et al., 2021b), comprising 778 patients. The flow chart of the study selection for this systematic review is shown in Figure 1. The characteristics and risk of bias of the included studies are shown in Table 1.

**TABLE 1:** Characteristics of included studies.

Author, year	Nation	Type of study	Sample size (T/C)	Patients	T	C	Age Mean (SD) or median [Range/IQR] (T/C)	Gender (male/Female)	Study period	Follow-up
Melikoglu, 2005 (Melikoglu et al., 2005)	Turkey	RCT	20/20	BD	Etanercept	Placebo	28.5 (5.3)/30.8 (6.2)	40/0	—	4 weeks
Tabbara, 2008 (Tabbara and Al-Hemidan, 2008)	Saudi Arabia	Retrospective	10/33	BU	IFX	TIS	26 [Range 16–35]/25 [Range 14–37]	—	—	At least 24 months
Yamada, 2010 (Yamada et al., 2010)	Japan	Retrospective	17/20	Refractory BU	IFX	TIS	37.6 (10.4)/(9)	32/5	1998–2008	6 months
Markomichelakis, 2011 (Markomichelakis et al., 2011)	Athens	Prospective	22 (19/8 eyes)	BU	IFX	TIS	30.5 (7.2)	14/8	—	4 weeks
Ma, 2014 (Ma et al., 2014)	China	Retrospective	19/35	Intestinal BD	Etanercept	TIS	37 (8.7)/40 (9.6)	34/20	—	At least 3 months
Vallet, 2015 (Vallet et al., 2015)	France	Multicenter retrospective	77/37	Severe and/or refractory BD	IFX	ADA	33.5 [IQR 28, 40]	57/57	2001–2013	21 [7–36] months
Fabiani, 2017 (Fabiani et al., 2017)	Italy	Multicenter retrospective	23/17	BU	ADA	ADA + TIS	41.9 (12)	22/18	—	12 months
Atienza-Mateo, 2019 (Atienza-Mateo et al., 2019)	Spain	Observational, open-label multicenter	103/74	Refractory BU	IFX	ADA	40.4 (10.1)/38.7 (1.3)	94/83	—	12 months
Yalçindag, 2020 (Yalçindag and Köse, 2020)	Turkey	Retrospective	20/33	Refractory BU	IFX	IFNα-2a	27.9 (4.2)/26.8 (6)	41/12	2010–2018	At least 12 months
De Simone, 2020 (De Simone et al., 2020)	Italy	Retrospective	7/15	Severe and refractory BU	IFX	IFNα-2a	29 (10)	14/8	2003–2019	30 ± 24 months
Kunimi, 2020 (Kunimi et al., 2020)	Japan	Retrospective	68/21	Refractory BU	IFX	ADA	—	—	2007–2019	—
Yang, 2021a (Yang et al., 2021a)	China	Retrospective	21/21	Refractory BU	ADA	TIS	29.4 (10)/27.5 (12.4)	23/19	2018–2019	At least 6 months
Yang, 2021b (Yang et al., 2021b)	China	Retrospective	21/24	BU	ADA	TIS	22.2 (15.3)/26.5 (10.5)	17/28	2015–2021	At least 6 months

T, test group; C, control group; SD, standard deviation; IQR: interquartile range; RCT, randomized controlled trial; BD, behçet’s disease; BU, behçet’s uveitis; IFX, inflfliximab; ADA, adalimumab; TIS, traditional immunosuppressant; IFNα-2a, interferon alpha-2a.

### 3.2 Study characteristics

Across all studies, 555 of 778 BD patients were followed for the effectiveness of subsequent therapies after exposure to at least one anti-TNF-α agent (except reference (Markomichelakis et al., 2011). The sample sizes were usually small and ranged from 22 to 177 patients. The male to female ratio was 1.5:1 (except reference (Tabbara and Al-Hemidan, 2008) and (Kunimi et al., 2020). The follow-up ranged from a minimum of 4 weeks to 36 [IQR 24, 72] months. The anti-TNF-α agent therapy group was treated with adalimumab (N = 214), infliximab (N = 302), and etanercept (N = 39). No studies focusing on golimumab or certolizumab.

A total of 201 patients were included in the control group. A subgroup review was conducted according to different control drugs, while the control group received traditional immunosuppressant (TIS) (N = 133), interferon alpha-2α (IFNα-2α) (N = 48) or placebo (N = 20). Per the study protocol, all studies were controlled clinical trials; among them were 1 randomized controlled trial (RCT) (7.7%), 1 prospective study (7.7%), and 10 retrospective studies (76.9%), and one multicentre open-label study (7.7%). Ten of thirteen studies (76.9%) were performed in the BU population, and the other study (7.7%) was conducted in the intestinal BD population. In the other two studies (15.4%), the subtype was undefined. Due to inconsistent outcome indicators, data that did not meet the requirements of the meta-analysis were systematically compared and reviewed according to the control drugs. We also performed a consolidation and meta-analysis of the small amount of homogeneous data. The risk of bias was assessed using the Cochrane Collaboration’s risk of bias tool (Supplementary Figure S1 and Supplementary Figure S2).

### 3.3 Effects of anti-TNF-α vs TIS for BU

**FIGURE 2:**
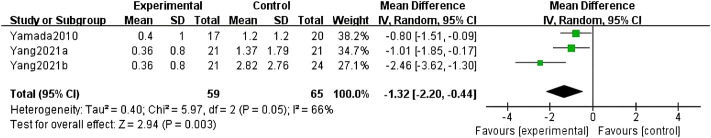
Forest plot of the relapse rate of Behçet’s uveitis in the anti-TNF-α- and traditional immunosuppressant-treated groups.

The systematic literature review retrieved 5 papers on the efficacy of anti-TNF-α versus TIS for BU, involving 189 participants. These studies provided consistent evidence for the prevention of relapse of BU. Four of these studies that involved 167 participants reported relapse rates. Combining three of these studies in a meta-analysis demonstrated a reduction in the relapse rates of participants with BU in the anti-TNF-α group compared with those in the TIS group (MD = -1.32, 95% CI: 2.20 to -0.44; *p* = 0. 003) (Figure 2) (Yamada et al., 2010; Yang et al., 2021a; Yang et al., 2021b). There was moderate heterogeneity in this result (I^2^ = 66%). Tabbara et al. (Tabbara and Al-Hemidan, 2008) proposed different modalities of measuring relapse rates, which resulted in the inability to pool their data in the meta-analysis. This study reached the similar conclusions that the mean number of relapses was significantly lower in the infliximab therapy group the TIS therapy group (average 1.2 [range, 0 to 4] vs average 6.3 [range, 4 to 7], *p* < 0.0001) and the infliximab group had a longer duration of remission (average, 17 months vs 5 months).

VA is another important outcome measure, and the five studies used different assessments in the comparison of anti-TNF-α therapy and TIS therapy. However, these outcomes are inconclusive. Yamada et al. (Yamada et al., 2010) reported the BCVA values at remission during the 6 months before and after the initiation of cyclosporin A (CsA) and infliximab in refractory BU. A total of 97% of the eyes in the infliximab group and 93% of the eyes in the CsA group exhibited visual improvement. However, there was no significant difference in the amount of improvement between these two groups. Markomichelakis et al. (Markomichelakis et al., 2011) conducted a 4-weeks, prospective, observational study of patients with panuveitis who received either an infliximab infusion or high-dose methylprednisolone intravenously or intravitreal triamcinolone acetonide at attack onset. The results of their study showed that the beneficial effects of the three treatment modalities on VA were comparable from baseline to the end of follow-up. The other two studies came from the same team, and one revealed that although inflammatory parameters (fluorescein angiography (FA) scores, −2 (−8, 0) points vs −8 (−14, −2.75) points, *p* = 0.002) (Yamada et al., 2010; Yang et al., 2021b) decreased more significantly in the adalimumab group in sight-threatening refractory BU, improvements in BCVA were similar in both groups. In another of their studies with naïve BU, the improvement in both FA scores (3.75 (−6.35 to −1.15) vs −12.85 (−15.61 to −10.08), *p* < 0.001) and BCVA (0.06 (−0.06−0.18) vs 0.33 (0.21−0.46) *p* = 0.024) in the adalimumab group was distinctly larger than that in the TIS group. At the same time, the difference became increasingly obvious after 5 months of treatment Table 2. There is another possible theory: Different lengths of follow-up may lead to differences and heterogeneity between studies. The a long-term follow-up in the study by Tabbara et al. (Tabbara and Al-Hemidan, 2008) show that the VA at the 24-months follow-up was significantly better in patients treated with infliximab than in those treated with conventional therapy (*p* = 0.006).

**TABLE 2:** The drug-related attributes of anti-TNF-α Agent.

Classifications	Structures	Administration routes	Originators (reference products)	Half-life (Jang et al., 2021)
Etanercept	sTNFR:Fc	Subcutaneous	Enbrel^Ⓡ^	3–3.5 days
Infliximab	Mouse-human chimeric mAb	Intravenous	Remicade^Ⓡ^	8–10 days
Adalimumab	Human mAb	Subcutaneous	Humira^Ⓡ^	10–13 days
Golimumab	Human mAb	Subcutaneous	Simponi^Ⓡ^	7–20 days
Certolizumab	PEGylated human Fab	Subcutaneous	Cimzia^Ⓡ^	2 weeks

### 3.4 Effects of anti-TNF-α vs IFNα-2α for BU

Comparing the anti-TNF-α group and IFNα-2α group, two studies involving 75 participants reported VA, MT, relapse, and clinical remission (Table 3). First, the conclusions of the two studies were similar, and there was no significant difference between the two biological agents in refractory BU. Second, pooling two studies in a meta-analysis demonstrated that MT decreased more significantly in patients treated with anti-TNF-α agents (MD = −8. 94, 95% CI: 16. 17 to -1. 70, *p* = 0. 02).

**TABLE 3:** Main outcomes of behçet’ uveitis in different studies

Author, year	T/C	VA, mean (SD)	MT (μm, mean (SD)	Relapse (times)	Response	AE (n/N)	SAE (n/N)
Melikoglu, 2005 (Melikoglu et al., 2005)	T (Etanercept)	—	—	—	—	2/20	—
	C (Placebo)	—	—	—	—	1/20	—
Tabbara, 2008 (Tabbara and Al-Hemidan, 2008)	T (IFX)	—	—	Mean [range]: 1.2 [0–4]	—	2/10	—
	C (TIS)	—	—	Mean [range]: 6.3 [4–7]	—	17/33	—
Yamada, 2010 (Yamada et al., 2010)	T (IFX)	—	—	Mean (SD): 0.4 (1.0)	Visual improvement: 97%	11/17	0/17
	C (TIS)	—	—	Mean (SD):1.2 (1.2)	Visual improvement: 93%	2/20	0/20
Markomichelakis, 2011 (Markomichelakis et al., 2011)	T (IFX)	VA, logMAR transformed: 0.5 (0.6)	—	—	—	0 (extraocular)	0 (extraocular)
	C (TIS)	VA, logMAR transformed: 0.7 (0.8)	—	—	—	0 (extraocular)	0 (extraocular)
Ma, 2014 (Ma et al., 2014)	T (Etanercept)	—	—	—	—	9/19	—
	C (TIS)	—	—	—	—	18/35	—
Vallet, 2015 (Vallet et al., 2015)	T (IFX)	—	—	—	CR: 44.6%	20/77	—
					PR: 51.8%		
	C (ADA)	—	—	—	CR: 56.5%	10/37	—
					PR: 43.5%		
Fabiani, 2017 (Fabiani et al., 2017)	T (ADA)	—	—	0 flares/100 patients/year	—	1/40	1/40
	C (ADA + TIS)	—	—	14 flares/100 patients/year	—		
Atienza-Mateo, 2019 (Atienza-Mateo et al., 2019)	T (IFX)	BCVA: 0.67 (0.34)	264.89 ± 59.74	Mean (SD): 1.13 ± 2.62	Remission: 76.47%	—	8/103
	C (ADA)	BCVA: 0.81 (0.26)	250.62 ± 36.85	Mean (SD): 1.66 ± 8.62	Remission: 82.43%	—	4/74
Yalçindag, 2020 (Yalçindag and Köse, 2020)	T (IFX)	VA, logMAR: 0.65 (0.14) at 12 months	194.7 ± 14 at 12 months	8 cases/12 months	Clinical remission (PR or CR): 80%	4/20	3/20
	C (IFNα-2a)	VA, logMAR: 0.37 (0.09) at 12 months	204 ± 12 at 12 months	21 cases/12 months	Clinical remission (PR or CR): 85%	33/33 (Flu-like syndrome)	2/33
De Simone, 2020 (De Simone et al., 2020)	T (IFX)	BCVA: 0.63 (0.43) at 12 months	234.2 ± 41.1 at 12 months	5 cases	CR: 71%	3/7	0/7
	C (IFNα-2a)	BCVA: 0.81 (0.25) at 12 months	233.7 ± 43.4 at 12 months	2 cases	CR: 80%	15/15 (Flu-like syndrome)	0/15
Kunimi, 2020 (Kunimi et al., 2020)	T (IFX)	VA, logMAR: 0.4 (0.77)	352 ± 235.4	Mean (SD): 0.03 ± 0.09/month	Eyes with improved VA: 52.7%	—	—
	C (ADA)	VA, logMAR: 0.36 (0.58)	296.6 ± 107.7	Mean (SD): 0.04 ± 0.06/month	Eyes with improved VA: 22.5%	—	—
Yang, 2021a (Yang et al., 2021a)	T (ADA)	BCVA change: 0.19 (0.25)	Change: 83.34 ± 156.74	Median [IQR]: 0 [0, 1]	—	7/21	—
	C (TIS)	BCVA change: 0.11 (0.21)	Change: 94.17 ± 218.43	Median [IQR]: 2 [0, 2.25]	—	2/21	—
Yang, 2021b (Yang et al., 2021b)	T (ADA)	BCVA mean change: 0.33	Change: 137.78 (mean)	Median [IQR]: 0 [0, 1]	—	11/21	—
	C (TIS)	BCVA mean change: 0.06	Change: 102.90 (mean)	Median [IQR]: 3 [1, 4.5]	—	7/24	—

T, test group; C, control group; VA, visual acuity; MT, macular thickness; AE, adverse events; SAE, serious adverse events; IFX, inflfliximab; TIS, traditional immunosuppressant; SD, standard deviation; logMAR, logarithm of the minimum angle of resolution; CR, complete response; PR, partial response; ADA, adalimumab; BVCA, best-corrected visual acuity; IFNα-2a, interferon alpha-2a.

### 3.5 Effects of anti-TNF-α vs placebo

The only RCT studied BD patients with mucocutaneous and articular manifestations (Melikoglu et al., 2005). Forty male patients with BD, all with positive pathergy and monosodium urate (MSU) tests and with mucocutaneous disease and/or arthritis, were randomized to receive either etanercept or placebo. After a 4-weeks observation period, etanercept proved to be effective in suppressing most of the mucocutaneous manifestations of BD, although the drug had no effect on the cutaneous response of MSU crystals.

### 3.6 Comparison of adalimumab and infliximab

Comparisons between adalimumab therapy and infliximab therapy (head-to-head design study) in other rheumatic diseases are common, whereas such a design for BU is rare. Three studies involving 380 participants reported the respective efficacy and safety in patients with BU. At the same time, a literature review revealed that both adalimumab and infliximab led to similar trends and qualitative conclusions (Vallet et al., 2015; Kunimi et al., 2020). That is, both adalimumab and infliximab are efficacious against BU. However, in an open-label multicentre study of adalimumab vs infliximab for BU refractory to conventional nonbiologic treatment, patients receiving adalimumab had significantly better outcomes in some parameters, including anterior chamber inflammation, vitritis, and BCVA. A nonsignificant difference was seen for macular thickness and the improvement in retinal vasculitis (Atienza-Mateo et al., 2019). We could not conduct a meta-analysis due to the use of a wide variety of outcome measures.

### 3.7 Adverse events

Thirteen studies reported the incidence of AEs. Six studies reported the occurrence of AEs in the anti-TNF-α therapy vs TIS therapy groups. Meta-analysis data from four studies combined showed that there was no statistically significant difference in AEs incidence between 2 groups (OR = 2.62, 95% CI: 0.52 to 13.15, participants = 167, *p* = 0.24). Two studies reported the occurrence of AEs in the anti-TNF-α therapy vs IFNα-2α therapy groups (De Simone et al., 2020; Yalçindag and Köse, 2020).Meta-analysis showed a significant difference in the AEs between the two groups (OR = 0.01, 95% CI: 0.00 to 0.08, *p* < 0.0001). Obviously, IFNα-2α treatment can cause systemic flu-like symptoms, and this might have contributed to the difference in results. If flu-like symptoms were excluded from AEs, there would be no such difference.

SAEs were not observed in seven of the studies. SAEs were reported in the other six studies, but the data could not be pooled for meta-analysis. Among all participants treated with anti-TNF-αtherapy, there were 22 cases of SAE as judged by the original authors or by us, severe infusion reaction (N = 4), pneumonia (N = 3), neoplasia (N = 3), lymphoma (N = 1), perianal abscess (N = 1), drug-induced lupus (N = 1), tuberculosis (N = 1), demyelinating disease (N = 1), allergic shock (N = 1), condyloma acuminata (N = 1), *mycobacterium avium* pneumonia (N = 1), severe oral ulcers (N = 1), palmoplantar skin reaction (N = 1), *escherichia coli* bacteremia (N = 1), and severe local reaction at the injection site (N = 1).

## 4 Discussion

Anti-TNF-α therapy for BD has a degree of consensus among real-world clinical experts. The recommendation of the European Alliance of Associations for Rheumatology (EULAR) in 2018 indicated that anti-TNF-α therapy could be used as a therapy in multiple complications of BD (Hatemi et al., 2018). We comprehensively searched the literature and included all clinical subtypes of BD complications. However, the fact remains that high-level evidence for efficacy and safety studies of anti-TNF-α therapy in BD is very limited, although policy makers and commissioners have long pointed out the need for a few definitive RCTs. Only one small, double-blind, randomized, placebo-controlled, 4-week follow-up study evaluated the efficacy of mucocutaneous manifestations in BD. In addition, there were one prospective studies, one multicenter open-label study and ten retrospective studies. Regarding the study population, ten of thirteen studies were performed in the BU population, and one other study was conducted in the intestinal BD population. In the remaining two studies the subtype was undefined. Therefore, this discussion of existing results is principally framed around BU.

Ocular complications occur in 30–70% of cases of BD, and the typical ocular involvement is chronic (Davatchi et al., 2016; Ksiaa et al., 2019), relapsing bilateral nongranulomatous uveitis that may involve anterior, posterior, intermediate or panuveitis, which results in vitritis, retinal infiltrates, sheathing of retinal veins, occlusive vasculitis, and macular oedema (Zeghidi et al., 2014; Ksiaa et al., 2019; Razumova and Godzenko, 2021). Although the prognosis is improving with the use of immunosuppressant therapy, 25% of patients with ocular disease become blind after treatment (Greco et al., 2018). First-line treatment of uveitis consists of local and/or systemic corticosteroids, and while these therapies are often highly effective, their chronic use can cause significant morbidity. These complications have led investigators to seek glucocorticoid free treatment. TNF-α, a cytokine produced primarily by monocytes, binds to two receptors: TNF receptor-1 or p55, a soluble receptor believed to be involved in proapoptotic and inflammatory pathways, and TNF receptor-2, a membrane-bound receptor that may regulate cell growth and proliferation (LaMattina and Goldstein, 2017; Tong et al., 2019; Karadag and Bolek, 2020). Both of these receptors are expressed in the vitreous, with mouse models also showing expression within the iris, ciliary body, and choroid. The anti-TNF-α agents include adalimumab, infliximab, golimumab, etanercept and certolizumab, The drug-related attributes are shown in. They block the interaction between TNF-α and both its soluble and membrane-bound receptors. The United States Food and Drug Administration and European Medicines Agency have approved some anti-TNF-α agents for the treatment of noninfectious uveitis. However, heterogeneity of the drug response still exists between different types of uveitis, and retinal vasculitis is a different condition. One hypothesis is that these patients who benefit most from anti-TNF-α either had isolated posterior segment inflammation (retinal vasculitis or cystic macular edema or papillitis) or panuveitis/uveoretinitis (rather than isolated anterior uveitis), preventing a poor outcome and requiring early intervention. Our meta-analysis showed that anti-TNF-α treatment reduced the relapse rate of BU compared with that after TIS therapy. However, there were no posttreatment differences in VA between the two groups. One probable reason is that the inclusion of patients with refractory uveitis resulted in an irreversible decline in baseline VA. Another possible reason is that the follow-up was too short to find a difference (Tabbara and Al-Hemidan, 2008). However, preventing the relapse of BD is crucial. Irreversible visual damage after repeated attacks is an important cause of blindness. We believe that visual benefits can be achieved by preventing relapse.

The exclusion of several single-arm clinical studies is highly warranted. There may be a bias effect caused by spontaneous recovery. Furthermore, steroid instillation or oral administration traditionally has been the initial step in the therapy of patients with BU, and the approach to treatment of BU has changed over time, becoming more aggressive with the earlier introduction of TIS agents, such as CsA, methotrexate, azathioprine, etc (Kump et al., 2008). This type of more economical traditional medicine can also achieve a comparable level of efficacy. Expensive biologics are also considered to be among, if not the main, drivers of the financial burden of autoimmune disease on patients and health-care systems. Hence, comparisons of their own set of advantages and disadvantages must be made with caution. Formal meta-analyses were performed, concluding that compared with TIS therapy, anti-TNF-α therapy effectively reduced the relapse of BU. In a qualitative descriptive analysis, these studies provided consistent evidence for the prevention of BU relapse. This is the most dominant contribution of this paper.

Comparisons between different targeted drugs (anti-TNF-α therapy and IFNα-2α therapy) are rare. In this study, we found that both IFNα-2α and infliximab were effective in inducing uveitis remission in patients with BD. However, there are minor differences in the degree of improvement MT. It is difficult to explain this discrepancy, and further studies are needed to better understand these results. Alternatively, this may simply be an incidental finding. In this study, we found the additional result that both adalimumab and infliximab provided similar types of trends and qualitative conclusions in two studies on BU. These head-to-head design comparisons may be common in other rheumatic diseases and are less common in BD.

Discussion and summary of the main results of our comprehensive search strategy: This was a comprehensive and systematic review summarizing the status of anti-TNF-α therapy among the myriad available treatment options. However, it is important to clarify some limitations that remain. The first limitation of this study is that the design of the controlled trial resulted in a tightening of the included literature, which was not sufficient for the meta-analysis. Second, the findings described here are affected by the limitations of the individual clinical trials that were selected, and most results were affected by significant heterogeneity. Third, we were unable to exclude publication bias. Based on the limitations mentioned, these negative and positive results all deserve further validation.

The above results indicate that, compared to TIS therapy, anti-TNF-α therapy effectively reduced the relapse of BU. This feature has important implications for preventing significant and permanent vision impairment or blindness. We call for caution in interpretation, as the evidence regarding anti-TNF-α therapy is very limited across the full spectrum of BD subtypes.

## Data Availability

The original contributions presented in the study are included in the article/Supplementary Materials, further inquiries can be directed to the corresponding authors.
