# Supplementary figures and images for: Anti-tumour necrosis factor-alpha agent therapy, compared with conventional therapy, reduces the relapse of uveitis in patients with behçet’s disease: A systematic review of controlled trials
Source: Front Pharmacol. 2022 Aug 19;13:912906. doi: 10.3389/fphar.2022.912906 (PMC9438790; doi:10.3389/fphar.2022.912906)

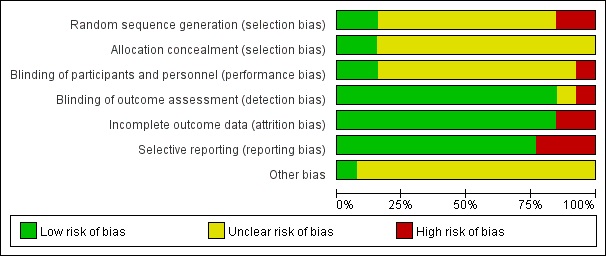

Supplement: Supplementary file 1 [file Image1.JPEG]

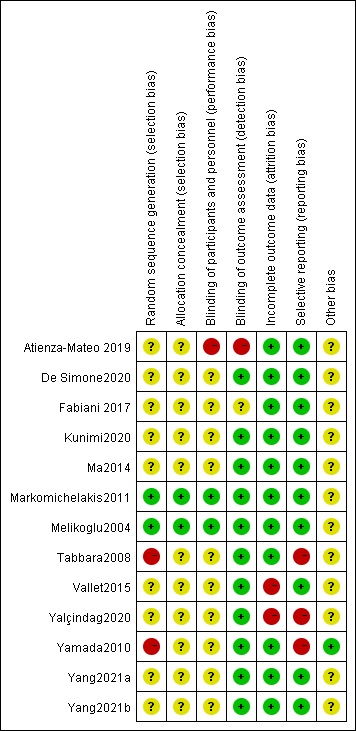

Supplement: Supplementary file 2 [file Image2.JPEG]
